# Draft Genome Sequence of *Pseudomonas* Strain P818, Isolated from Glyphosate-Polluted Soil

**DOI:** 10.1128/genomeA.01079-13

**Published:** 2013-12-19

**Authors:** Gaoyi Cao, Yunjun Liu, Guiming Liu, Jianhua Wang, Guoying Wang

**Affiliations:** Institute of Crop Sciences, Chinese Academy of Agricultural Sciences, Beijing, Chinaa; Department of Agronomy, Tianjin Agricultural University, Tianjin, Chinab; CAS Key Laboratory of Genome Sciences and Information, Beijing Institute of Genomics, Chinese Academy of Sciences, Beijing, Chinac; College of Agriculture and Biotechnology, China Agriculture University, Beijing, Chinad

## Abstract

*Pseudomonas* strain P818 was isolated from glyphosate-polluted soil in China. This bacterium presents a capacity for high glyphosate tolerance. We present the draft genome sequence of the strain *Pseudomonas* P818. The genes involved in the glyphosate tolerance were identified. This genomic information will facilitate the study of glyphosate tolerance mechanisms.

## GENOME ANNOUNCEMENT

A number of species, especially microbes, may develop mechanisms to survive when they are under continuous stress in extreme environments (1, 2). Generally, some pivotal genes in specific pathways may first appear or be changed in the effort to adapt (3). Glyphosate-induced stress to microbes is common in glyphosate-polluted soil. The microorganisms that are tolerant of glyphosate can acquire this tolerance through two approaches: first, through the overexpression of 5-enolpyruvylshikimate-3-phosphate synthase (EPSPS) to resist the inhibition of the shikimate pathway, and second, through the degradation of glyphosate to eliminate glyphosate toxicity (4–8). The key genes involved in the response of glyphosate resistance can be identified and characterized in further research.

*Pseudomonas* strain P818 was isolated from glyphosate-polluted soil around an aged chemical company in China. This bacterium can grow well in M9 minimal medium containing 300 mM glyphosate and shows an eximious capacity for high glyphosate tolerance. The genome of P818 was sequenced, and the pivotal genes associated with glyphosate resistance and salinity stress tolerance were identified. This work provides useful information for the development of transgenic glyphosate-tolerant plants.

The *Pseudomonas* P818 genome was sequenced by a whole-genome shotgun strategy using Illumina HiSeq 2000. Genomic libraries of 180 bp, 800 bp, and 3 kb were constructed and sequenced, providing about 500-fold coverage of the genome. *De novo* assembly was performed using SOAP*denovo*2 (9), resulting in 35 (defined as >500-bp) scaffolds. The gaps inside the scaffolds were closed with local assembly of the reads with one end located in the gap region and its paired read well-aligned on the contigs. The remaining gaps were closed with Sanger sequencing of PCR products by primer walking. Protein-encoding genes were predicted by combining the results of Glimmer 3.02 (10) and ZCurve (11), followed by manual inspection. tRNA and rRNA genes were identified by tRNAscan-SE (12) and RNAmmer (13), respectively. Functional annotation was performed by searching against the NCBI nr, Swiss-Prot (14), InterProScan (15), COG (16), and KEGG (17) databases.

The draft genome sequence of *Pseudomonas* P818 is composed of 5,100,938 bases, with a G+C content of 63.4%. It contains 4,732 open reading frames (ORFs), 4 rRNA operons, and 58 tRNA genes. There are 3,640 genes involving 22 COG function categories and 2,663 genes annotated into 2,172 KEGG orthologous groups by KAAS, which are involved in 173 metabolic pathways.

### Nucleotide sequence accession number.

This whole-genome shotgun project has been deposited in GenBank under the accession no. ATKM00000000.
